# Editorial Note: Significant increase in eco-efficiency of China’s grain production from 2000 to 2022: Trend changes, typological evolution, and driving factors

**DOI:** 10.1371/journal.pone.0359242

**Published:** 2026-09-28

**Authors:** The PLOS One Editors

The *PLOS One* Editors issue this Editorial Note to inform readers that PLOS has concerns about the article’s [1] compliance with PLOS policy on Authorship, and that Reference 4 appears to be irrelevant to this article [1].

The corresponding author stated that Reference 4 was cited to provide mechanistic background for the broader assertion that ecological stresses can affect food production. The Editors do not consider this matter resolved.
